# The effect of osseodensification and different thread designs on the dental implant primary stability

**DOI:** 10.12688/f1000research.17292.1

**Published:** 2018-12-05

**Authors:** Abdullah Saleh Almutairi, Maher Abdullatif Walid, Mohamed Ahmed Alkhodary

**Affiliations:** 1 Department of Periodontology, College of Dentistry, Qassim University, Buraidah, Qassim, 51452, Saudi Arabia; 2 Department of Prosthetic Dental Science, College of Dentistry, Qassim University, Buraidah, Qassim, 51452, Saudi Arabia; 3 Department of Prosthodontics, Faculty of Dentistry, Alexandria University, Alexandria, Egypt

**Keywords:** Implant primary stability, osseodensification, implant thread designs, Periotest.

## Abstract

**Background** **:** It is difficult to achieve good primary stability of dental implants in soft bone, such as that in the posterior maxillae. Osseodensification (OD) burs, working in a non-subtractive fashion, condense the implant osteotomy bone in lateral direction and increase in the bone implant contact. Also, dental implants with deeper threads, and decreased thread pitch can increase initial bone implant anchorage.

**Methods**: This study utilized 48 custom-made machined surface dental implants that were 13 mm long, with a major diameter of 4.5 mm and a minor diameter of 3.5 mm, a thread pitch of 1 mm, a thread depth of 0.5 mm, and a 4 mm long cutting flute at the apex.  The implants were divided into 4 groups, each group was made of 12 implants with a different thread design; V-shaped, trapezoid, buttress, and reverse buttress. The implants were inserted in 4-mm thick cancellous bone slices obtained from the head of Cow femur bone. The ostoetomies were prepared by conventional drilling and by OD drilling. Each inserted implant was then tested for primary stability using the Periotest. The Periotest values (PTVs) for the implant stability were tabulated and analyzed using a chi square test at significance level p< 0.05.

**Results** **:** The results of this this study revealed no statistically significant difference between the Periotest readings for the implants in each category placed in either the OD or the regular osteotomies. However, it has been found that the implants placed in regular drilling ostoetomies had a significantly better primary stability than the implants placed in OD osteotomies.

**Conclusions** **:** It was concluded that OD is not necessary in situations where there is bone of good quality and quantity.

## Introduction

The primary stability of dental implants depends on the quantity and quality of the available bone, the implant macro- and micro-design, the implant surface features, and the surgical technique used for creating the osteotomy. Conventionally, ostoetomies are created with bone-removing drills, and the last drill had a smaller diameter than that of the implant to ensure primary stability. However, this technique is only effective up to certain limits in soft bone, such as that in the posterior maxillae ^1^.

Osseodensification (OD) burs, working in a non-subtractive fashion, condense the implant osteotomy soft bone in lateral direction, leading to a greater bone volume and density, an increase in the bone implant contact, with subsequent increase in insertion torque levels, and reduction in micromotion ^2– 6^. However, it has been claimed that OD increases the implant bone bed density, but does not improve implant primary stability ^7^.

Another maneuver to increase implant primary stability in a poor bone quality situation, is to use an implant with deeper threads, and decreased thread pitch, to increase initial bone implant anchorage. This principle can be applied to different dental implant thread designs; V-shaped, buttress, reverse-buttress, and trapezoid. However, each thread design is thought to give a varying degree of apical and lateral compression to the surrounding bone, which will produce a certain amount of osteocompression and primary stability ^8– 13^.

Clinically, the dental implant primary stability can be evaluated using several techniques, such as the amount of torque needed during insertion, or after insertion using the resonance frequency analysis technology implemented in the Osstell device, or the mechanical percussion principle used in the Periotest ^14– 20^.

Although the OD may minimize the use of other more invasive techniques, such as ridge splitting ^21^, sinus lifting ^22^, and onlay bone grafting ^23^, there has been a claim that the OD surgical technique may not be effective in improving the primary stability of dental implants ^7^, and that the dental implant macro-design is not crucial for the implant primary stability so long as the surrounding bone is of good quantity and quality ^24^. The aim of this study was to test the effect of both variables, the OD surgical technique and the implant macro-design, on the dental implant primary stability, using custom made dental implants with four different thread shapes, with the same thread pitch and depth, placed in two different types of ostoetomies prepared by the conventional and the OD technique, and evaluated using the Periotest.

## Methods

### Implants

**Figure 1.**
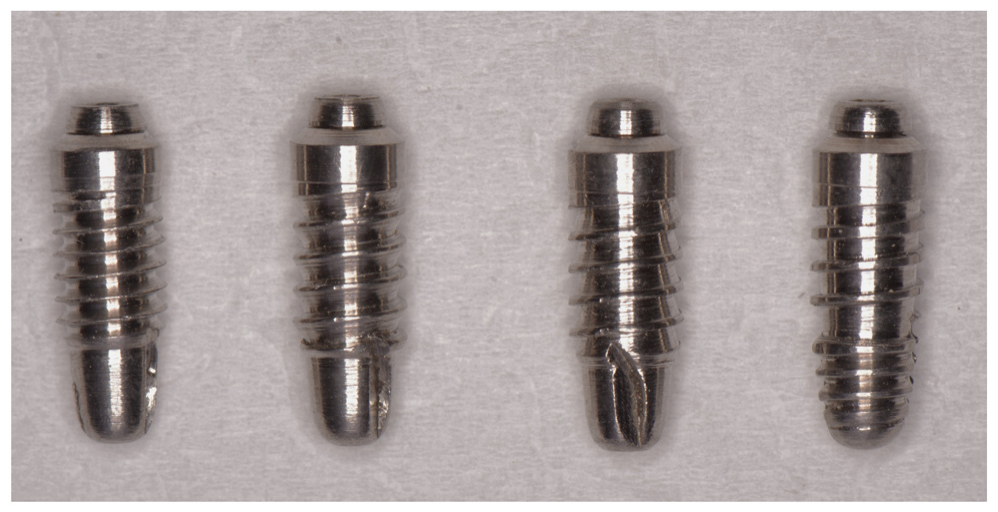
The custom-made dental implants used in this study. The thread shapes, from left to right, are V-shaped, buttress, reverse buttress, and trapezoid.

This study utilized 48 custom made machined surface dental implants that were 13 mm long, with a major diameter of 4.5 mm and a minor diameter of 3.5 mm. The implants were divided into four groups, each group made up of 12 implants with a different thread design: V-shaped, trapezoid, buttress, and reverse buttress ( Figure 1). All the groups had the same thread pitch of 1 mm, a thread depth of 0.5 mm, and a 4 mm long cutting flute at the apex of the implants.

### Bone source and drilling

**Figure 2.**
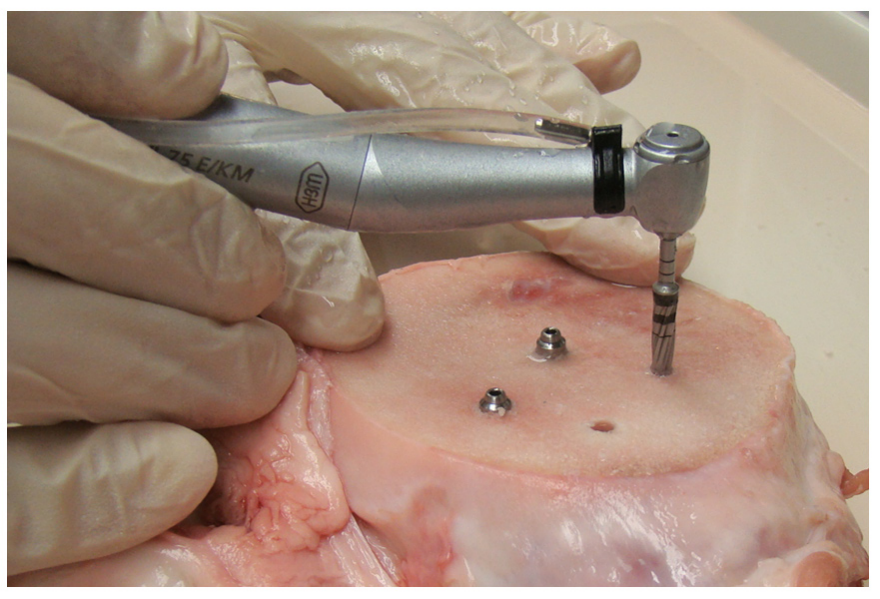
Drilling the osseodensification osteotomy using the densah bur after placing the first implant in regular drilling osteotomy.

**Figure 3.**
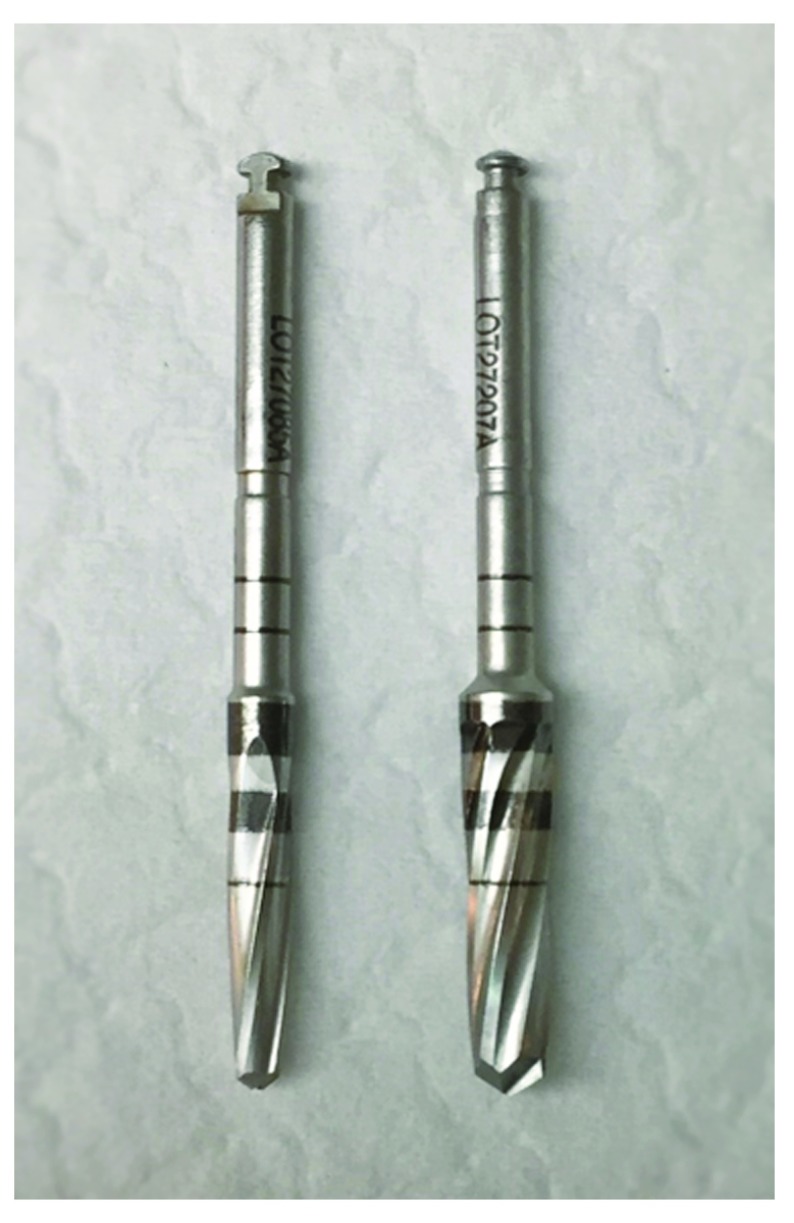
The osseodensification Densah burs.

The head of a Cow femur was used as the bone model ^25^, to reveal its cancellous bone core, it was sliced in 4 cm thick slices in which the implants were inserted. As shown in Figure 2, all the implants were inserted into osteotomies prepared by drills coupled to hydrated hand piece of a dental drill unit (Osseoset 200, Nobel Biocare) to a full length of 13 mm. In total, six implants of each group were inserted into osteotomies prepared by conventional cutting drills (cutting mode, clockwise rotation 1100 RPM), starting with drill size 1.5 mm, 2 mm, 2.4–2.8 mm, 2.8–3.3mm, 3.2–3.6mm and 3.8–4.2mm. The other six were inserted into osteotomies prepared by OD burs (Densah Burs) (OD mode, contra-clockwise rotation 1100 RPM), that started first with conventional drilling with 1.5 mm and 2 mm cutting drills (cutting mode), then OD burs ( Figure 3) of size 2.5 mm (DENSAH Bur-G2 VS2228), 3.0 mm (DENSAH Bur-G2 VT2535), and 3.5 mm (DENSAH Bur-G2 VS3238) were used.

### Stability testing

**Figure 4.**
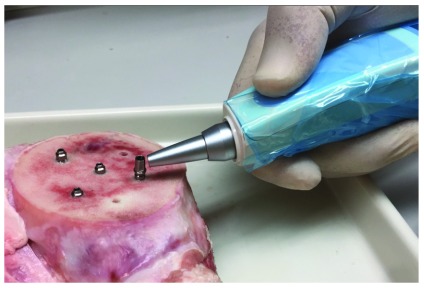
Using the Periotest to evaluate the dental implant primary stability, the Periotest tip is applied to the abutment.

Each inserted implant was tested for primary stability using the Periotest, with the tip of the Periotest retractable pin was applied to the same position in the implant abutment ( Figure 4). The Periotest values interpretation, as described by the manufacturer, state that values between -8 and zero indicate good primary stability, and values above that range indicate insufficient integration between the implant and the surrounding bone.

### Statistical analysis

The Periotest values (PTVs) for the implant stability were tabulated and analyzed using statistical package for social science (SPSS version 20 for windows). Comparisons between the study groups were carried out using the chi square test at significance level p< 0.05

## Results

### Comparison of implant types

This work assessed the effect of OD versus regular drilling on dental implant primary stability. To rule out the effect of the implant design, the most commonly used thread shapes were tested in both ostoetomies. The Periotest was used to read each implant primary stability. The results of this this study revealed no statistically significant difference between the Periotest readings for the implants in each category placed in either the OD or the regular osteotomies ( Table 1).

**Table 1.** Comparison of the Periotest values for primary stability of the different implant threads placed in osseodensification (OD) and regular ostoetomies.

Thread design	Drilling method	Periotest values for each implant insertion						Mean	SD	P
		First	Second	Third	Fourth	Fifth	Sixth			
V-shaped	Regular	-7	-4.1	-5.5	-7.5	-7.4	-6.9	-6.4	1.33	0.512
	OD	-8	-7.6	-6.1	-6.2	-6.2	-6.9	-6.8	0.81	
Trapezoid	Regular	-5.1	-6.5	-7.8	-7.7	-7.5	-6.9	-6.9	1.02	0.076
	OD	-7.2	-6.8	-5.3	-4.4	-5.1	-4.6	-5.6	1.16	
Buttress	Regular	-7.7	-6.6	-8	-8	-7.7	-7.7	-7.6	0.51	0.06
	OD	-8	-6.9	-5.9	-5.8	-5.3	-7.8	-6.6	1.12	
Reverse-buttress	Regular	-7.3	-6.3	-7.2	-7.1	-7.4	-7.2	-7.1	0.39	0.121
	OD	-8	-7.2	-6	-5.6	-5.8	-4.9	-6.3	1.13	

### Comparison of regular and OD ostoetomies

When all the implants placed in regular drilling ostoetomies were compared to all the implants placed in OD ostoetomies, statistical analysis of the Periotest readings for primary stability has shown that the implants placed in regular drilling ostoetomies were significantly more stable than the implants placed in OD osteotomies ( Table 2).

**Table 2.** Comparison of the Periotest values for primary stability of the all the implant placed in osseodensification (OD) versus those placed in regular ostoetomies.

Drilling method	Mean	Std. deviation	n	P value
Regular	-7.00	0.95	24	0.026
OD	-6.31	1.11	24	

## Discussion

The aim of this study was to evaluate the effect of dental implant osteotomy preparation, using the OD technique, on the dental implant primary stability compared to conventional drilling. Since the dental implants may present many variables, such as the thread designs and surface treatments, which may affect the primary stability, this study utilized custom-made implants, with machined surface to avoid the effect of surface treatments on primary stability, and different thread patterns having the same thread pitch and depth, to detect which thread design will provide better primary stability in conjunction with OD.

Since the implants used in this study were custom-made, it was difficult to use the resonance frequency analysis to evaluate the primary stability of the implants as the Osstell device requires the use of a smart peg which was difficult to custom make; however, the Periotest was used. Javed *et al.* ^14 ^ have stated that the both the Osstell and the Periotest can be used to measure the dental implant primary stability, although the Periotest readings are less sensitive. Andresen *et al.* ^16^ have also approved the use of Periotest once the clinicians consider its limitations and the difficulty in results interpretation. A different perspective was given by Noguerol *et al.* ^17 ^who stated that the Periotest mechanical testing would definitely give a better evaluation for implant stability than any radiographic study. Furthermore, Oh *et al.* ^18, 19^ found that the Periotest was comparable and as reliable as the Osstell. However, Aparicio *et al.* ^20^ emphasized that it is important to consider several readings of either device over a long period of time in order to be able to evaluate the implant stability.

In the presence of soft bone, under-sizing the implant osteotomy is thought to give a better implant primary stability; however, Jimbo *et al.* ^1^ stated that this technique is efficient when the implant bed is decreased by 10% of its diameter and that any further decrease did not improve the primary stability. Huwais and Meyer ^2^ introduced the bone compaction technique through the OD drilling, and claimed that it increased the insertion torque, bone-to-implant contact, and accordingly resulted in greater primary stability compared to conventional drilling and to Summers ^15^’ osteotome technique. This hypothesis has been confirmed by the work of Lahens *et al.* ^3^ who reported a significantly higher bone-to-implant contact for OD, and Lopez *et al.* ^4^ who tested the OD technique *in vivo* and reported its significant success over conventional drilling mechanically using the pull-out testing and microscopically using the histomorphometry. Additionally, Trisi *et al.* ^5^ have shown that OD allows the use of wider implants diameters in narrow edentulous ridges, with consequent increase in bone volume. This increase in bone was later shown to reach 30% of the original ridge dimensions by Podaropoulos ^6^.

However, the results of this study did not find any statistically significant difference between the effects of OD and conventional drilling on the dental implant primary stability with any of the different thread designs used. This came in agreement with the findings of Wang *et al.* ^7^, who reported that OD increased the apparent density of the peri-implant bone, but did not significantly improve the bone-implant contact, or the primary stability, and that OD created high strains at the bone implant interface, with damage to the bone trabeculae leading to extended periods of resorption and delayed secondary stability.

In accordance with the findings of Abuhussein *et al.* ^8^, the implants used in this study had deep threads with a decreased thread pitch to ensure bone anchorage, and based on the conclusion of Chong *et al.* ^11^, being without self-tapping properties, the threads were thought to provide higher primary stability than self-tapping threads. However, none of the tested thread shapes has shown any superiority in achieving better primary stability. 

 Notably, the results of this study showed that there was no statistically significant difference between the OD and the regular drilling techniques, nor between the different thread designs used based on the Periotest values recorded for the implant primary stability. Considering the bone model used, Alkhodary *et al.* ^25^ have stated that the elastic modulus of the cancellous bone of the cow femur head was comparable to that of the cancellous bone of the human mandible, which is in turn more compact than the bone in the posterior maxillae, and according to Chong *et al.* ^11^ and Summers ^15^, optimal bone quality and quantity can mask any difference in the implant different designs. This suggestion has been further potentiated by Bischof *et al.* ^24^ who studied the factors affecting the dental implants primary stability, and reported that it is not the diameter or length of the implant, nor the implant thread deepening that affect primary stability, rather than the bone type in the mandible or the maxilla.

Accordingly, it can be concluded that OD is not useful in compact bone, and might have a different effect in soft bone, and that the effects of different thread designs are more noticed in cancellous, rather than compact bone as shown by Kong *et al.* ^10^ who reported different stress distribution patterns by the different thread shapes at the cancellous bone-implant interface. This came in agreement with the second finding of this study, where all implants placed in regular osteotomies had a significantly better primary stability than all the implants placed in OD osteotomies. This can be explained by the fact that soft bone has wider marrow spaces between the bone trabeculae, allowing for bone compaction, rather than the compact bone which the OD would lead to lateral compression that exceeds the viscoelastic limit of the thick and dense bone trabeculae, with subsequent damage and a weaker bone implant interface. Also, it is recommended to examine the effect of OD in an *in vivo* situation, where the bone available is soft, and where the monitoring process by the Periotest, or the resonance frequency analysis can be conducted several times to detect the effect of OD on both the primary and secondary stability of the dental implants.

## Data availability

All data underlying the results are available as part of the article and no additional source data are required.
