# Supplementary material for: The effect of osseodensification and different thread designs on the dental implant primary stability
Source: F1000Res. 2018 Dec 5;7:1898. [Version 1] doi: 10.12688/f1000research.17292.1 (PMC6518436; doi:10.12688/f1000research.17292.1)
Supplement: Peer review history

# Peer review: Reviewer response for version 1

**DOI:** 10.5256/f1000research.18905.r47949

**Published:** 2019-05-14

**Authors:** Zachary Evans

**Affiliations:** 1 Department of Stomatology, College of Dental Medicine, Medical University of South Carolina, Charleston, SC, USA

This is a focused manuscript attempting to evaluate the effects of thread design on implant stability with an OD drill protocol in trabecular bone. It is a surprise that the OD protocol did not improve initial implant stability. Therefore, this is clinically relevant as it may influence providers to use the OD protocol for ridge expansion or sinus lifting, but not for densification. Studies like this are giving us information that may influence our clinical decisions in this regard. It is a simple, but very useful study that I hope will lead to a larger clinical study. 

 Two small comments to consider:

1. Consider the use of the animal model terminology "bovine" vs. "Cow". 
2. Consider adding a comment on clinical relevance to the discussion - for example, what clinical scenarios would be appropriate or not for the OD protocol.  

I have read this submission. I believe that I have an appropriate level of expertise to confirm that it is of an acceptable scientific standard.

# Peer review: Reviewer response for version 1

**DOI:** 10.5256/f1000research.18905.r45551

**Published:** 2019-03-29

**Authors:** Jun Lee

**Affiliations:** 1 Department of Oral and Maxillofacial Surgery, School of Dentistry, College of Dentistry, Wonkwang University, Iksan, South Korea

This paper appears to be a comparison of densah bur and conventional drill system according to implant design in animal models. Overall, the content is simple but well proven. Only in this experiment, it is considered that the animal model is designed properly.

 I mention a few minor revisions: 

1. Abstract is 4-mm thick cancellous bone slices, but it is written as 4-cm in the text.
2.  In the experimental design, four implants are placed in one Cow femur slice, and I wonder how much distance there is between the implants (critical distance between implants). Indeed, the important consequences of this experiment would be to provide an experimental condition that does not interfere with drilling or implant placement to yield reliable results

I have read this submission. I believe that I have an appropriate level of expertise to confirm that it is of an acceptable scientific standard.

# Peer review: Reviewer response for version 1

**DOI:** 10.5256/f1000research.18905.r43178

**Published:** 2019-01-23

**Authors:** Khalid Almas

**Affiliations:** 1 Department of Preventive Dental Sciences, College of Dentistry, Imam Abdulrahman Bin Faisal University, Dammam, Saudi Arabia

This is an interesting in vitro study. Osseodensification technique is getting popularity due to improved local bone quality for implant placement with the technique. 

 The pilot work is interesting as far as the unified approach is concerned with 13 mm long implants and customized surface configurations. It is suggested that in future following variations should be considered to test the effect of osseodensification.

 1. Implants length less than 13 mm (8-11 mm)

 2. Variable width.

 3. Different thread pitch and depth.

 4. Implant surface characteristics should be with SLA surface or any other non-machined surface.

 5. The immediate stability of implants may not reflect the normal healing and stability physical equilibrium. As in real time clinical implant surgery, the initial stability and after 1-2 weeks stability may vary.  

 The discussion part of the manuscript should include variables mentioned above and future studies may include those points. 

 Further, radiographic or any other imaging technique should be used to see immediate bone-implant contact in both regular and OD technique.

I have read this submission. I believe that I have an appropriate level of expertise to confirm that it is of an acceptable scientific standard, however I have significant reservations, as outlined above.
